# Using Wannier functions to improve solid band gap predictions in density functional theory

**DOI:** 10.1038/srep24924

**Published:** 2016-04-26

**Authors:** Jie Ma, Lin-Wang Wang

**Affiliations:** 1 Joint Center for Artificial Photosynthesis and Materials Sciences Division, Lawrence Berkeley National Laboratory, Berkeley, California 94720, USA

## Abstract

Enforcing a straight-line condition of the total energy upon removal/addition of fractional electrons on eigen states has been successfully applied to atoms and molecules for calculating ionization potentials and electron affinities, but fails for solids due to the extended nature of the eigen orbitals. Here we have extended the straight-line condition to the removal/addition of fractional electrons on Wannier functions constructed within the occupied/unoccupied subspaces. It removes the self-interaction energies of those Wannier functions, and yields accurate band gaps for solids compared to experiments. It does not have any adjustable parameters and the computational cost is at the DFT level. This method can also work for molecules, providing eigen energies in good agreement with experimental ionization potentials and electron affinities. Our approach can be viewed as an alternative approach of the standard LDA+U procedure.

Density functional theory (DFT)^1^ is the main working horse for material simulations, especially for ground-state properties such as atomic structures and binding energies. However, it is well known that the DFT, in particular the Kohn-Sham eigen energy^2^, significantly underestimates band gaps. This is related to the lack of derivative discontinuity in the exchange-correlation (XC) energy when the total number of electrons crosses an integer point^3^,^4^. Over the years, various methods have been developed to overcome this deficiency. One popular approach is the hybrid functional^5^,^6^,^7^, which mixes the exact exchange with local/semilocal XC functionals. Although widely successful, these methods depend on the mixing parameters and are computationally more expensive than local/semilocal functionals such as the local density approximation (LDA) or generalized gradient approximation. An even higher-level method is the GW and the related random-phase approximation (RPA)^8^,^9^, but the high computational cost of RPA makes it only applicable to small systems^10^. There exists another approach to correct the DFT and its related Kohn-Sham Hamiltonian: the Koopmans’ theorem^11^,^12^,^13^,^14^,^15^,^16^,^17^,^18^,^19^,^20^,^21^,^22^. The original Koopmans’ theorem only states that in Hartree-Fock, the first ionization energy is equal to the highest occupied orbital energy if the wave function relaxations are ignored. In the literature^11^,^12^,^13^,^14^,^15^,^16^,^17^,^18^,^19^,^20^,^21^, the terminology “Koopmans’ theorem” was used to indicate the straight-line condition (SLC) of the total energy *E*(*n*) as a function of the continuous number of electrons *n* between two integer points^23^. However, the LDA *E*(*n*) curve is convex^24^,^25^. As derived by Janak^26^ as well as Yang *et al*.^24^,^25^, within a local or semilocal exchange correlation functional or a generalized Kohn-Sham calculation (e.g., including the explicit exchange integral), for a *N*-electron system, 
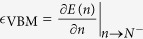
, 
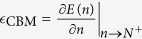
 with 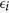 being the eigen energies of the valence band maximum (VBM) and conduction band minimum (CBM), so the lack of the derivative discontinuity and the convexity of the LDA energy leads to an underestimation of the band gap 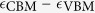. One explicit way^15^ to enforce the SLC is to modify the LDA total energy to


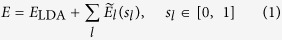


with 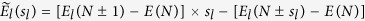 (+ for adding electrons to unoccupied orbitals, and − for removing electrons from occupied orbitals). *E*_*l*_(*N* ± *s*_*l*_) is the self-consistent-field LDA energy after adding/removing *s*_*l*_ electrons on the *ϕ*_*l*_ orbital. Note 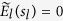 for *s*_*l*_ = 0 or 1. The resulting *E* of Eq. (1) should be a straight line within 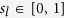. Taking the variational minimum of *E* with respect to *ψ*_*i*_ (here *s*_*l*_ = |〈*ϕ*_*l*_|*ψ*_*i*_〉|^2^ is the projection of *ψ*_*i*_ on *ϕ*_*l*_), we obtain a modified Kohn-Sham equation


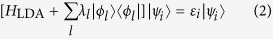


with 
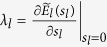
, providing corrections to eigen energies.

The original SLC applies only to eigen orbitals *ϕ*_*l*_. For molecules and atoms, the eigen energies in Eq. (2) yield accurate quasi-particle energies^13^,^14^,^15^,^16^, and the SLC makes the VBM and CBM eigen energies equal to the LDA total-energy differences *E*(*N*) − *E*(*N* − 1) and *E*(*N* + 1) − *E*(*N*) (the so-called ΔSCF method^27^,^28^,^29^). To be consistent with the terminology for solids, for molecules, we also call the highest occupied molecular orbital (HOMO) and the lowest unoccupied molecular orbital (LUMO) the VBM and CBM, respectively. Correcting the Hamiltonian and eigen energies of the Kohn-Sham equation has many advantages, e.g. having correct band alignments for transport calculations. However, the above procedure fails for extended systems such as solids, because adding/removing electrons on an extended eigen state only gives an infinitesimal local charge-density change, which leads to 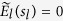 and *λ*_*l*_ = 0 (no correction). In ref. 30, the authors proposed adding/removing a finite number of electrons in the primitive cell, which improves the LDA band gaps. However, an empirical parameter is needed to adjust the amount of electrons to be added/removed.

In this work, we propose a new approach that extends the original SLC and makes it applicable to solids. The basic ansatz is that the energy curve *E*_*l*_(*N* ± *s*_*l*_) for removing/adding *s*_*l*_ fractional electrons on any single-particle orbital *ϕ*_*l*_ (not necessarily eigen states) in the valence-band/conduction-band subspace should be a straight line. It removes the condition that *ϕ*_*l*_ must be an eigen state. The linear dependence of the total energy with respect to *s*_*l*_ in Eq. (1) can be motivated by a derivation with a Hartree-Fock formalism, which is similar to the derivation of the original Koopmans’ theorem (Supplementary Note). From such a derivation, it is clear that the enforcement of the SLC via Eq. (1) implies the removal of the self-interaction error of *ϕ*_*l*_, which is expected to improve the band gaps^31^. Furthermore, following the path of ref. 23, one can also attempt to construct a grand canonical ensemble expression for the total energy *E*(*N* ± *s*_*l*_), using *N* + 1 or *N* − 1 many body wave functions that contain or exclude the single particle orbital *ϕ*_*l*_ as expressed in Supplementary Note. In refs 29,32,33, the author proposed a general orbital dependent variant functional with a SLC compliant form. Both a set of minimizing orbital and canonical (eigen) orbital was used. In a self-consistent solution, for some functionals they tested, there was a localization force to localize the minimizing orbital, making it Wannier like. It is also known that in the self-interaction correction formalism^34^, the orbital can be well localized. All these make it plausible to assume the orbital *ϕ*_*l*_ can be a localized wave function within the valence (or conduction) band manifold, rather than canonical eigen states. We like to emphasize that these arguments are only used to show the plausibility of Eq. (1), providing some insights and motivations, instead of giving a rigorous derivation. In this work, we can treat Eq. (1) just as an ansatz. To introduce nonzero corrections, we need localized *ϕ*_*l*_. The Wannier functions (WF)^35^,^36^ are the most localized orbitals within the valence-band and conduction-band subspaces. Hence we will use WF as *ϕ*_*l*_ in Eqs (1) and (2). The WFs are mutually orthogonal, and the collective WFs for valence/conduction band fill in the valence/conduction band subspace. We will show that the resulting eigen energies are in excellent agreement with experiments. This approach can also be viewed as an alternative approach of the popular LDA+U method^37^ where the self-interaction energy of the localized orbital *ϕ*_*l*_ is removed. Although there are self-consistent approach to calculate the U parameter in the LDA+U method^38^, in practice U is often used as a fitting parameter. However, in our approach, no parameters will be used. In terms of the computational cost, after *λ*_*l*_ is calculated, the computational cost for applying a wave function to the Hamiltonian Eq. (2) is similar to that of LDA+U, which both include the calculation of projections. The cost of calculating *λ*_*l*_ is similar to that of a defect calculation using a supercell, as will be discussed below.

## Results

### Calculating *λ*_*l*_

In the calculation of *λ*_*l*_, we find that the screening from other electrons plays an important role. If we remove WFs from the charge density and perform non-self-consistent calculations, the band gap corrections can be overestimated by several eV. To include the screening effect, we must calculate *E*_*l*_(*N* ± *s*_*l*_) self-consistently. To add/remove fractional WF *ϕ*_*l*_ in the spin-up channel, we variationally optimize all the spin-up states under the constraint of the orthogonality to *ϕ*_*l*_, and optimize the spin-down states in the conventional way, to minimize the total energy *E*_*l*_(*N* ± *s*_*l*_), which is


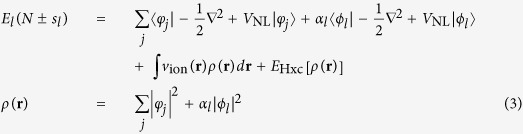


where *V*_NL_ is the nonlocal-potential operator, *v*_ion_(**r**) is the ionic potential, and *E*_Hxc_ is the conventional Hartree and LDA XC energy. For valence-band WFs, *α*_*l*_ = 1 − *s*_*l*_ and the summation of *j* is from 1 to *N*/2 − 1 (*N* is an even number) for the spin-up channel, and 1 to *N*/2 for the spin-down channel; for conduction-band WFs, *α*_*l*_ = *s*_*l*_, and the summation is from 1 to *N*/2 for both spin-up and spin-down channels. In both the +*s*_*l*_ and −*s*_*l*_ cases, the *φ*_*j*_ in the spin-up channel is required to be orthogonal to *ϕ*_*l*_, i.e., 〈*φ*_*j*_|*ϕ*_*l*_〉 = 0. Using a Lagrangian multiplier for this constraint, the minimization of *E*_*l*_(*N* ± *s*_*l*_) with respect to *φ*_*j*_ (while *ϕ*_*l*_ is kept fixed) yields


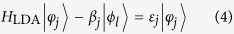


with *β*_*j*_ = 〈*ϕ*_*l*_|*H*_LDA_|*φ*_*j*_〉 for the spin-up channel and *β*_*j*_ = 0 for the spin-down channel. The conventional conjugate-gradient formalism can be used to solve the above equation and obtain the minimum *E*_*l*_(*N* ± *s*_*l*_). Note, when *s*_*l*_ = 0, Eq. (3) returns to the conventional DFT expression; for finite *s*_*l*_, a uniform background compensation charge is used for solving the Poisson equation, similar to a charged defect calculation. A few *s*_*l*_ (at least three *s*_*l*_) need to be calculated to obtain *λ*_*l*_. To exclude the interactions between the WF and its images, we perform the *λ*_*l*_ calculation with spin-polarization using a supercell equal to 4 × 4 × 4 times primitive cell. To test the convergency of the *λ*_*l*_ with respect to the supercell size, we have calculated the *λ* for the Si VBM state (Si *p*-state) in the 4 × 4 × 4, 5 × 5 × 5, and 6 × 6 × 6 supercells. The results are shown in Supplementary Fig. S1. The energy difference of *λ* between the 4 × 4 × 4 and 6 × 6 × 6 supercells is 45 meV. If we fit the *λ* values to *A* + *B*/*L* (*A* and *B* are fitting parameters and *L* is the supercell length), the *λ* is ~0.52 eV as *L* tends to infinity. Thus, the estimated error in the 4 × 4 × 4 supercell for the Si VBM is ~0.1 eV. We have also calculated the error for the Si CBM state. The energy difference of the *λ* for the CBM state between the 4 × 4 × 4 and 6 × 6 × 6 supercells is 43 meV, which is similar to that for the VBM state. Thus, we may estimate that the error in the 4 × 4 × 4 supercell due to the finite supercell size is ~0.1 eV for both the CBM and VBM states.

After *λ*_*l*_ is obtained, in principle, one should self-consistently solve Eq. (2). However, we have tested several bulk system (e.g., GaAs), and the self-consistent effect for the band gap correction is rather small (see discussions below). Here we just use the original LDA wave-function *ψ*_*i*_, and take the expectation value of Eq. (2), which is similar to the G_0_W_0_ flavour. Thus the modified eigen energy of the original LDA eigen state *ψ*_*i*_ can be accurately calculated as:


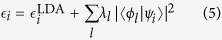


with 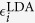 being the original LDA eigen energy.

In the above procedure, we always keep the WF *ϕ*_*l*_ fixed. As mentioned above, after *λ*_*l*_ is obtained, one can solve Eq. (2) and yield new *ψ*_*i*_ and thus construct new WF *ϕ*_*l*_. However, one major feature of the current approach is that Eq. (2) will keep the origin subspace of the valence band and conduction band subspaces of the LDA Hamiltonian. This is because 〈*ψ*_*c*_|*ϕ*_*l*_〉〈*ϕ*_*l*_|*ψ*_*v*_〉 = 0; thus the *λ*|*ϕ*_*l*_〉〈*ϕ*_*l*_| term will not mix the valence bands with conduction bands. Between these two subspaces, the current procedure is a “scissor” operator without mixing them. As a result, if we use the maximal localized WFs, the *ϕ*_*l*_ in the new iteration should be the same as that in the previous iteration because the subspace is not changed. One could, however, raise an issue about fixing *ϕ*_*l*_ in Eq. (4) when *s*_*l*_ electrons is removed or added on *ϕ*_*l*_. For example, will {*φ*_*j*_, *ϕ*_*l*_} of Eq. (4) form the valence (conduction) band subspace of a single-particle Hamiltonian? This is discussed in the Supplementary Note. Right now we use a fixed *ϕ*_*l*_ during removing or adding electrons as a part of the ansatz. In the future, it might be interesting to test the effect of varying *ϕ*_*l*_, perhaps under a variational form using both the eigen states and localized states as described in refs 29,32,33.

### Band energies for solids

**Figure 1:**
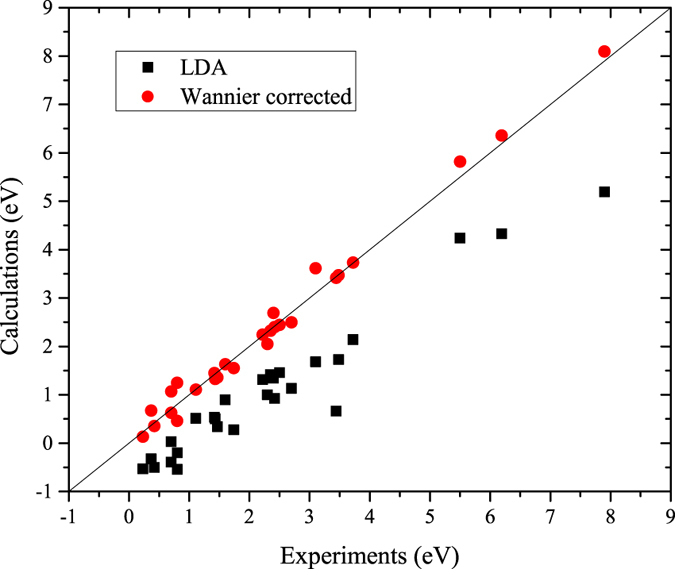
The calculated LDA and Wannier-corrected band gaps versus the experimental band gaps^39^ for the 27 solids. The LDA calculations significantly underestimate the band gaps, and the Wannier-corrected results are in good agreement with experiments.

We have calculated 27 semiconductor compounds (Supplementary Table S1), including conventional semiconductors and oxides with experimental band gaps ranging from 0.2 to 8 eV, covering a wide range of physical situations and application interests. The Wannier-corrected band gaps along with the LDA band gaps are plotted in Fig. 1 versus the experimental band gaps^39^. Our LDA band gaps (Supplementary Table S1) agree well with previous published results. They significantly underestimate the experimental values (even negative for some compounds), and there is no simple correlation between the LDA and experimental band gaps. The Wannier-corrected band gaps are in good agreement with experiments, and the errors are on par with the more-expensive GW method^40^. In the following, we discuss in more details for a few examples.

GaAs is a representative of main-group semiconductors, and it has a band gap of 1.43 eV experimentally. The LDA band gap is 0.5 eV after considering the spin-orbit coupling (SOC). As stated in Methods, we construct one *s* and three *p* projected WFs at the As/Ga site for the valence/conduction bands. The VBM state is purely contributed from the As-*p* projected WFs, i.e. 
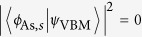
 and 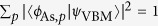, so the VBM energy correction equals to *λ*_As,*p*_ = −0.58 eV. The CBM energy correction is purely from the Ga-*s* projected WF, i.e., 
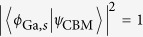
, with *λ*_Ga,*s*_ = 0.25 eV. Therefore, the Wannier-corrected band gap is 0.5 + 0.58 + 0.25 = 1.33 eV, which agrees well with experiments. At the bottom of the valence bands, the Γ_6*v*_ (Γ_1*v*_ without SOC) state is purely contributed from the As-*s* projected WF, and the energy correction is *λ*_As,*s*_ = −0.88 eV, which is 0.3 eV more than the correction to the VBM. The LDA bottom valence band is 12.84 below the VBM, so the Wannier-corrected value is 13.14 eV, which agrees excellently with the experimental value of 13.1 eV^41^.

ZnO is a prototypical transition-metal oxide with a band gap of 3.4 eV experimentally. However, its LDA band gap is only 0.66 eV. There are controversies for whether the GW can reproduce the experimental band gap^42^,^43^. The typical hybrid functional also gives a too small band gap (2.5 eV)^44^. For the valence bands, we construct five Zn-*d* projected, one O-*s* projected, and three O-*p* projected WFs. Due to the strong *p-d* hybridizations in ZnO, both the O-*p* and Zn-*d* projected WFs contribute to the VBM, with the energy corrections of −1.1 eV and −0.9 eV, respectively. For the CBM, similar to the GaAs case, only the Zn-*s* projected WF contributes, and the energy correction is 0.75 eV. As a result, the Wannier-corrected band gap is 3.41 eV, in excellent agreement with experiments. We note that a major part of the large correction comes from the *d* component in the VBM. For II-VI systems with weaker *p-d* hybridizations, e.g. ZnS, the *d* projected WFs only contribute ~0.2 eV to the VBM correction and the LDA band gap errors tend to be smaller than that of ZnO.

**Figure 2:**
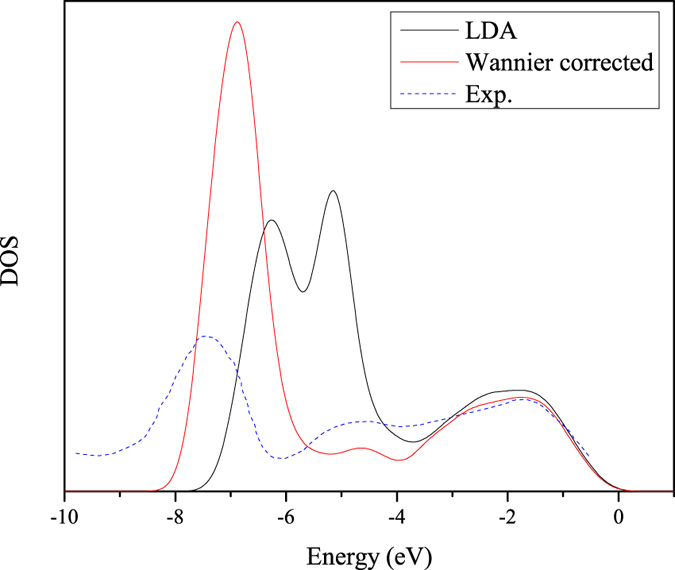
The DOS of ZnO from the LDA and Wannier-corrected calculations, and the experimental curve (blue dashed line) taken from ref.^39^. Because the cross section is not considered in the DOS calculations, the height of the experimental curve should not be compared with the calculations. The peak position and shape of the Wannier-corrected DOS are significant improved, compared to the LDA DOS.

Another interesting quantity in ZnO is the energy position of the Zn 3 *d* bands. The density of states (DOS) calculated by the LDA and the new method along with the experimental results are plotted in Fig. 2. The experimental Zn 3 *d* peak is ~7.5 eV below the VBM^45^. The LDA peaks are ~2 eV higher compared to experiments. The Wannier-corrected peak is ~7 eV below the VBM, which agrees with the GW results and is better than the hybrid functional results^44^,^46^. Furthermore, in experiments, Zn 3 *d* is a single peak, while LDA yields two peaks and the low-energy peak couples strongly with the O 2 *p* states^44^. Our Wannier-corrected method reduces the *p-d* repulsion, and merges the two peaks into a single one in agreement with experiments.

The above discussion indicates that the Wanner-corrected method not only improves band gaps, but also improves band energies inside valence bands. The same is true for conduction bands. Table 1 shows that the conduction-band energies at the Γ, X, and L points are all corrected well by our method, for two most widely-studied semiconductors: Si and GaAs.

### Ionization potentials and electron affinities for molecules

The above results show that the new method works well for solids. One remaining question is whether it also works for molecules. In molecule calculations, we use the open-boundary condition for the Poisson equation to avoid image interactions^47^. For atoms or simple molecules such as LiCl, the projected WFs are just the eigen orbitals, so our method behaves the same as the ΔSCF method and agrees well with experiments^28^.

**Figure 3:**
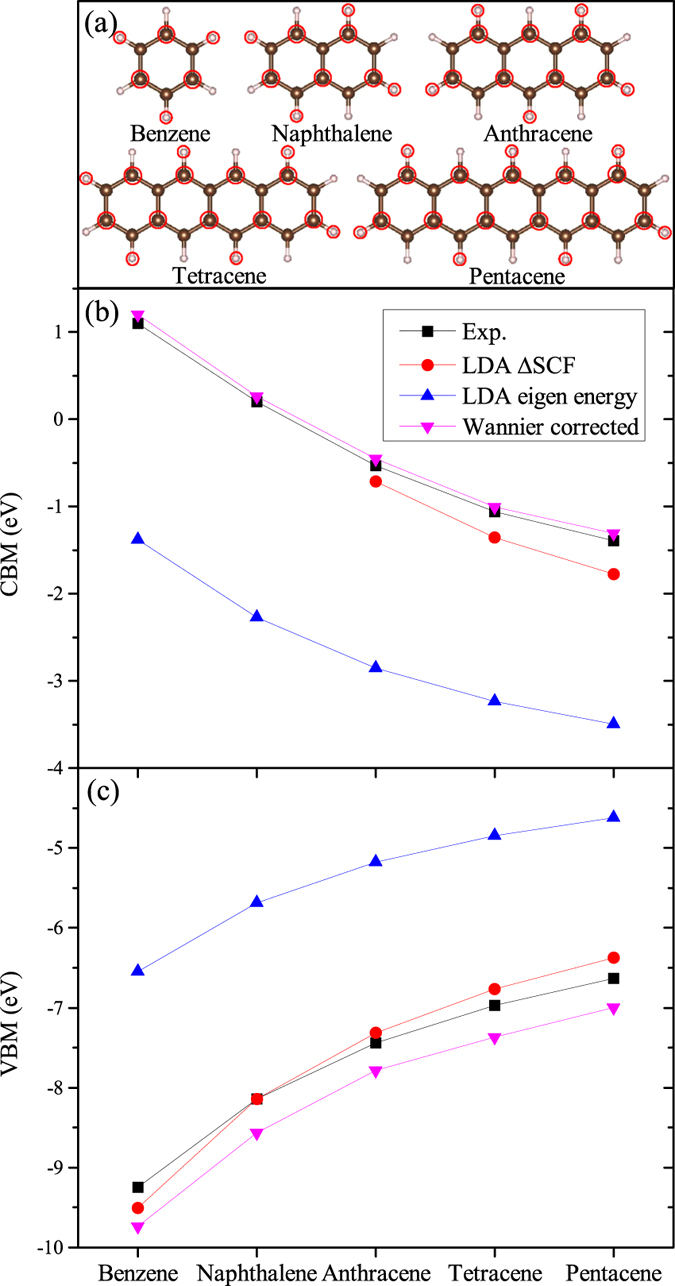
The structures of the calculated molecules (**a**), the experimental data^48^,^49^, ΔSCF energies, LDA eigen energies, and Wannier-corrected eigen energies for the CBM (**b**) and the VBM (**c**). For these molecules, the Wannier-corrected eigen energies show significant improvement upon LDA, and agree well with the experimental ionization potentials and electron affinities.

Next we discuss a more complex molecule serial: polycyclic aromatic hydrocarbons, shown in Fig. 3(a). As the number of benzene-ring increases, the molecules will eventually become a 1D system. We symmetrically pick half of the carbon and hydrogen atoms [circled by red in Fig. 3(a)], and construct C-*s*, C-*p*, and H-*s* projected WFs on these atom sites. Other choices have been tested and the results are similar [to keep the symmetry, the WFs at the atoms uncircled in Fig. 3(a) can also be included in Eq. (2), with an overall factor of 1/2 applied to all *λ*_*l*_]. In these cases, the WFs are no longer the eigen orbitals. The VBM of these molecules are purely contributed from the C-*p*_*z*_ projected WFs constructed by the occupied orbitals, and the CBM are purely contributed from the C-*p*_*z*_ projected WFs constructed by the unoccupied orbitals. Figure 3 plots the experimental vertical ionization potentials (IP) and the vertical electron affinities (EA)^48^,^49^, the LDA eigen energies, the ΔSCF energies, and our Wannier-corrected results. The Wannier-corrected results agree excellently with the experimental EAs, and are only ~0.4 eV lower than the experimental IPs, showing significant improvement compared to the LDA eigen energies. Although the ΔSCF energies also agree well with the experiments (note for benzene and naphthalene, the EAs are above vacuum, so *E*(*N* + 1) cannot be calculated), the energy trend of the new method is much better. As the molecular size increases, the ΔSCF energy errors (compared to experiments) for both the EAs and IPs keep increasing, and eventually the ΔSCF energies will converge to the LDA eigen energies for the extended 1D system (the increase of the error is nevertheless slow, so the ΔSCF works for moderate-sized molecules). However, the errors of our method do not increase with the molecule size. It indicates that the new method works from small molecules all the way to extended solids, and thus it is a general approach for electronic structure calculations. It is interesting to notice the small difference between the ΔSCF results and the Wannier-corrected results for small molecules (e.g., benzene). It indicates the band gap correction is not so sensitive to the exact degree of localization. It explains why our method is insensitive to the specific choice of WFs and also explains why the ΔSCF method works for mediate size molecules shown in Fig. 3.

## Discussion

We have presented a new method to calculate electronic structures for solids. This method is based on an extended SLC, and uses WFs as localized orbitals for the fractional electron addition/removal. It does not have any adjustable parameters and is computationally much cheaper than hybrid functionals or GW calculations. For solids, it yields accurate eigen energies not only for band-edge states but also for other states inside the bands. It also works for molecules, yielding good IPs and EAs. Our method does not depend sensitively on the XC functionals. Through our work, we have used the LDA functional. We also find that the GGA functionals give very similar results. Hybrid functionals (e.g., HSE) which already partially correct the band gaps (thus a less convex *E*_*l*_(*s*_*l*_) curve) will simply have a smaller 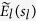 in Eq. (1) and hence a smaller *λ*_*l*_ correction in Eq. (2). We also find that the final results do not sensitively depend on the exact choice of the WFs (see Methods section).

It is interesting to compare our approach with the LDA+U method. The LDA+U method tries to construct a Hubbard model for the localized states (e.g., *d* or *f* atomic orbitals). It thus uses the atomic orbitals as *ϕ*_*l*_. It keeps the Hamiltonian rotationally invariant among the five/seven oniste *d*/*f* states. The *d*/*f* states are often partially occupied or unoccupied, resulting in different prefactors to their *λ*_*l*_ of Eq. (2) (depending on their occupations), but all proportional to the same U parameter. In our approach, the occupied and unoccupied Wannier functions are treated differently with their own *λ*_*l*_ values and there is no rotational invariance between the occupied and unoccupied Wannier functions. Our approach is a “scissor” operator between occupied and unoccupied states without mixing them, whereas LDA+U will mix occupied and unoccupied states. While the LDA+U is aimed at improving energies of localized states, not necessarily to improve band gaps especially for main-group semiconductors, our approach improves the band gaps for main-group semiconductors (*s* and *p* states), as well as the eigen energies of the localized *d* states. We note that LDA+U was recently used to treat common semiconductors^50^,^51^. However, the conventional LDA+U and its popular implementation has an ambiguity for the relatively arbitrary nature of *ϕ*_*l*_, which is often taken as the atomic orbital.

In our procedure, the *λ* values for all the WFs need to be calculated by self-consistent calculations. It might be possible to speed up such calculations by approximation methods. For example, Poilvert *et al*.^52^ have suggested using a fixed ratio for all the conduction (valence) bands between their self-consistent screened and non-self-consistent unscreened values. Such approximation might be certainly possible. For example, in a defect supercell calculation, perhaps the *λ* values for the WFs far away from the defect can take the value from the bulk calculation. Further tests are needed in this regards.

## Methods

The DFT calculations are performed by the PEtot code^47^ within the LDA. Norm-conserving pseudopotentials are used. The plane-wave energy cutoff and *k*-point mesh guarantee the convergence of the eigen energies within 0.01 eV. For the LDA band gap calculations, we use the primitive cells with the lattice parameters and atomic coordinates fully optimized. The SOC is considered.

The WFs are constructed using the Wannier90 code^53^. We construct the WFs for valence bands and conduction bands separately, by projecting the eigen states onto the atomic orbitals^54^,^55^. The atomic orbitals are chosen based on their predominance in the valence (conduction) bands. For example, for GaAs, the valence-band WFs are constructed by projecting the valence bands onto one As *s* and three As *p* orbitals; the conduction-band WFs are constructed by projecting the conduction bands onto one Ga *s* and three Ga *p* orbitals. The resulting WFs (Supplementary Fig. S2) are all highly localized. However, we would like to emphasize that the choice of the atomic orbital projected WFs is merely a convenience provided by the Wannier90 code and the final results do not sensitively depend on the specific choice of the WFs. For example, we have compared the final band gaps using the above projected WFs and the maximally-localized WFs, and the resulting band-gap corrections differ by less than 0.01 eV.

In our method, to get each *λ*_*l*_, we need two more self-consistent static calculations in a 4 × 4 × 4 supercell besides the ground-state energy. Taking GaAs as an example, to correct the band gap, we need two *λ*_*l*_: one for occupied *p* states and the other for unoccupied *s* states. Thus, in total we need four self-consistent calculations in the 4 × 4 × 4 supercell (the ground state energy can be calculated in the primitive cell). Each of such calculations has the cost of a charged defect calculation using a supercell. Note, there are Wannier functions at all atom sites in the supercell, but their *λ* are the same because of the translational symmetry. The procedure is of course slower than LDA calculations, but it is much faster than the GW calculations. In practice, we also find it faster than the hybrid functional calculations for solids. Moreover, because all the self-consistent calculations are independent, we can easily parallelize them on supercomputers.

## Additional Information

**How to cite this article**: Ma, J. and Wang, L.-W. Using Wannier functions to improve solid band gap predictions in density functional theory. *Sci. Rep*. **6**, 24924; doi: 10.1038/srep24924 (2016).

## Supplementary Material

Supplementary Information

## Figures and Tables

**Table 1:** The band gaps (eV) measured from the VBM to the conduction bands at the Γ, X, and L points of Si and GaAs.

Compound	Conduction band	LDA	Wannier-corrected	Experiment
Si	Γ_15c_	2.58	3.21	3.34
	Γ_2′c_	3.53	4.09	4.15
	X_1c_	0.63	1.2	1.13
	L_1c_	1.58	2.17	2.04
	L_3c_	3.33	3.96	3.91
GaAs^a^	Γ_1c_	0.5	1.33	1.43
	Γ_15c_	3.82	4.67	4.72
	X_1c_	1.32	2.14	2.18
	X_3c_	1.52	2.33	2.58
	L_1c_	0.90	1.73	1.85

Besides the band gap, the new method also improves other eigen energies inside the conduction bands.

^a^In ref. 41, the single group notation was used, so we average the SO-splitted energies of the Γ_15c_ state.
