# Supplementary material for: Using Wannier functions to improve solid band gap predictions in density functional theory
Source: Sci Rep. 2016 Apr 26;6:24924. doi: 10.1038/srep24924 (PMC4845067; doi:10.1038/srep24924)
Supplement: Supplementary Information [file srep24924-s1.pdf]

Supplementary Information: Using Wannier functions to improve solid band gap predictions in density functional theory

Jie Ma and Lin-Wang Wang

Joint Center for Artificial Photosynthesis and Materials Sciences Division, Lawrence Berkeley National Laboratory, Berkeley, California 94720, USA

Supplementary Figure

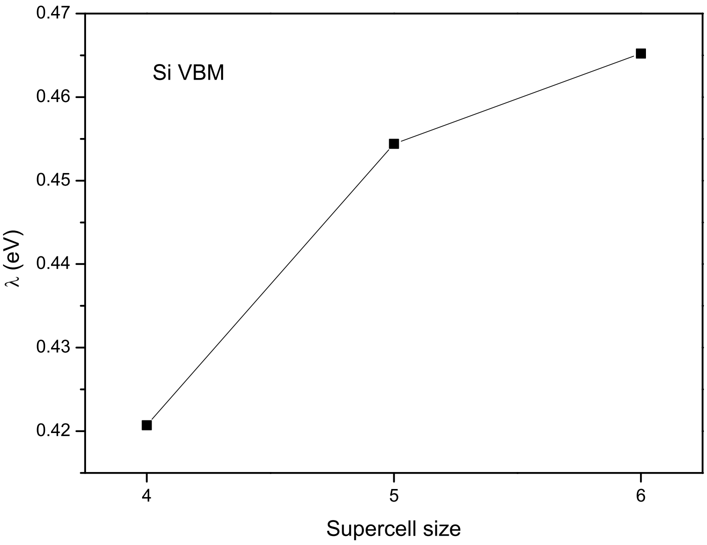

Supplementary Figure S1: The energy correction (λ) for the Si VBM state in the $4 \times 4 \times 4$, $5 \times 5 \times 5$, and $6 \times 6 \times 6$ supercells. The energy difference between the $4 \times 4 \times 4$ and $6 \times 6 \times 6$ supercells is 45 meV. If we fit the data to $A + B/L$ (A and B are fitting parameters and L is the supercell length), the energy correction is 0.52 eV, as L tends to infinity. Thus the error of λ and the resulting eigen energy in the $4 \times 4 \times 4$ supercell is ~ 0.1 eV. We find the same for the conduction band.

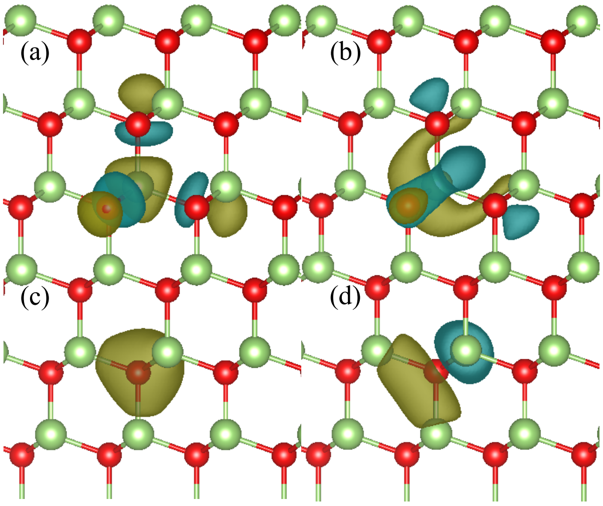

Supplementary Figure S2: The conduction-band Ga-s (a) and Ga-p projected (b), and valence-band As-s (c) and As-p projected (d) Wannier functions in GaAs. The red and green balls represent As and Ga atoms. The yellow and blue colors represent the positive and negative isovalues of the Wannier functions, respectively. The Wannier functions are strongly localized. They form a complete and orthogonal basis set for the conduction-band/valence-band subspace.

Supplementary Note

In the Hartree-Fock (HF) method, the many body wave function is expressed as a Slater determinant

$$\Psi_N = \frac{1}{\sqrt{N!}} \begin{vmatrix} \phi_1(\mathbf{r}_1) & \phi_2(\mathbf{r}_1) & \cdots & \phi_N(\mathbf{r}_1) \\ \phi_1(\mathbf{r}_2) & \phi_2(\mathbf{r}_2) & \cdots & \phi_N(\mathbf{r}_2) \\ \vdots & \vdots & \ddots & \vdots \\ \phi_1(\mathbf{r}_N) & \phi_2(\mathbf{r}_N) & \cdots & \phi_N(\mathbf{r}_N) \end{vmatrix} \tag{S1}$$

Here the $\{\phi_i\}$ are orthonormal single-particle orbitals, but not necessarily eigen orbitals because a unitary transform of the orbitals does not change the slater determinant. The HF total energy can then be expressed as:

$$\begin{aligned} E_{\mathrm{HF}}(N) &= <\Psi_N|H|\Psi_N>= \sum_i h_i + \frac{1}{2}\int d\mathbf{r}d\mathbf{r}'\frac{\rho(\mathbf{r})\rho(\mathbf{r}')}{|\mathbf{r}-\mathbf{r}'|} \\ &- \frac{1}{2}\sum_{i,j}\int d\mathbf{r}d\mathbf{r}'\frac{\phi_i(\mathbf{r})\phi_j^*(\mathbf{r})\phi_i^*(\mathbf{r}')\phi_j(\mathbf{r}')}{|\mathbf{r}-\mathbf{r}'|} \end{aligned} \tag{S2}$$

Here $\rho(\mathbf{r}) = \sum_i |\phi_i(\mathbf{r})|^2$ is the total charge density, and $h_i =< \phi_i(\mathbf{r})| - \frac{1}{2}\nabla^2 - \sum_R \frac{Z_R}{|\mathbf{R}-\mathbf{r}|}|\phi_i(\mathbf{r})>$. It is impossible to write down a $N \pm s_l$ electron many body wave function for fractional s_l except in a grand canonical ensemble as will be discussed later. However, intuitively, to describe a orbital ϕ_l which is partially occupied by $1 - s_l$ electrons (in below we will only discuss removing s_l electrons from the ϕ_l orbital for simplicity), it makes sense to replace ϕ_l by $\sqrt{1-s_l}\phi_l$ in Eq. (S2), and $\rho = \sum_{i\neq l}|\phi_i|^2 + (1-s_l)|\phi_l|^2$. We find that the self interaction energy in the Coulomb term cancels out with that in the exchange term. As a result, we have

$$E_{\mathrm{HF}}(N - s_l) = E_{\mathrm{HF}}(N-1)|_{\mathrm{without}\ \phi_l} + (1-s_l)E_l \tag{S3}$$

Here $E_l = \langle\phi_l|H_{\mathrm{HF}}|\phi_l\rangle$. H_{HF} is the HF single-particle Hamiltonian, and E_l is the expectation value of H_{HF} by the ϕ_l orbital. Note, ϕ_l does not need to be a HF eigen orbital. In the derivations above, just like in the orignial Koopmans' theorem, we assume $\{\phi_i\}$ do not change with s_l.

The important point of Eq. (S3) is that, $E_{\mathrm{HF}}(N - s_l)$ is a linear function of s_l. The nonlinear term (which only exists in the self Coulomb interaction and exchange interaction terms under the frozen orbital approximation) has been cancelled out. If we take $s_l = 0$ and ϕ_l to be the VBM state, Eq. (S3) is the original Koopmans' theorem. The Eq. (S3) can be applied to arbitrary orbital ϕ_l in the occupied subspace, and it shows that without the self-interaction term, $E_{\mathrm{HF}}(N - s_l)$ should be a linear function of s_l. The same is true for the unoccupied subspace.

The plausibility of applying the straight-line energy condition to Wannier functions can also be viewed from the a grand canonical ensemble point of view for the total energy $E(N \pm s_l)$, similar to that in Ref. [S1]. In Ref. [S1], the authors constructed a grand canonical ensemble, which is a statistical mixture of N-electron pure states Ψ_N and $(N+1)$-electron pure states Ψ_{N+1}. For the state with $N + s_l$ electrons [$s_l \in (0,1)$], the probability of Ψ_N is $1 - s_l$ and that of Ψ_{N+1} is s_l, respectively. According to the variational principle, the lowest energy of the ensemble is $(1-s_l)E_N + s_l E_{N+1}$, where E_N and E_{N+1} are the ground state energies of the N-electron and $(N+1)$-electron pure states respectively [S1]. Thus the total energy is a linear function of s_l. To extend it to the Wannier functions, we can construct a similar grand canonical ensemble with Ψ_N and constrained $\Psi^{\rm c}_{N+1}$ (we first discuss the conduction band). To ensure a Wanner function ϕ_l is fully contained in the constrained $(N+1)$-electron wave function $\Psi^{\rm c}_{N+1}$ (here ϕ_l is within the conduction band subspace and thus orthogonal to Ψ_N, i.e. $\int d\mathbf{r}_1 \phi_l^*(\mathbf{r}_1)\Psi_N(\mathbf{r}_1, \cdots, \mathbf{r}_N) = 0$), the constraint on $\Psi^{\rm c}_{N+1}$ is

$$\int d\mathbf{r}_1 d\mathbf{r}'_1 d\mathbf{r}_2 \cdots d\mathbf{r}_{N+1} \phi_l^*(\mathbf{r}'_1)\Psi^{\rm c}_{N+1}(\mathbf{r}'_1, \mathbf{r}_2, \cdots, \mathbf{r}_{N+1})\Psi^{\rm c*}_{N+1}(\mathbf{r}_1, \mathbf{r}_2, \cdots, \mathbf{r}_{N+1})\phi_l(\mathbf{r}_1) = \frac{1}{N+1} \quad \text{(S4)}$$

which indicates the probability of electrons on ϕ_l is one (i.e., the Wannier function is fully occupied). One can use $\Psi^{\rm c}_{N+1}$ that satisfies Eq. (S4) to variationally minimize the total energy. We may call the resulting energy $E^{\rm c}_{N+1}$ the constrained ground-state energy. According to the variational principle, the statistic total energy of the grand canonical ensemble with $N + s_l$ electrons is $(1-s_l)E_N + s_l E^{\rm c}_{N+1}$. It shows that after a fractional-electron addition onto a Wannier function (constructed within the conduction band subspace), the total energy follows a linear function of s_l. Similarly, to remove s_l electrons from a Wannier function ϕ_l (constructed within the valence band subspace), we require a constraint on the $(N-1)$-electron wave function $\Psi^{\rm c}_{N-1}$:

$$\int d\mathbf{r}_1 \phi_l^*(\mathbf{r}_1)\Psi^{\rm c}_{N-1}(\mathbf{r}_1, \mathbf{r}_2, \cdots, \mathbf{r}_{N-1}) = 0 \quad \text{(S5)}$$

which ensures that the Wannier function is not contained in $\Psi^{\rm c}_{N-1}$. The statistical total energy of the constrained grand canonical ensemble $(1-s_l)E_N + s_l E^{\rm c}_{N-1}$ once again shows the linearity with s_l.

There could be an issue for the self-consistent calculations of the Wannier functions after s_l electrons are removing from the Wannier function ϕ_l. If a Wannier function is partially occupied and the rest of the wave functions are solved self-consistently, one issue is whether the rigid ϕ_l is still within the valence band subspace of the new $N - s_l$ electron Hamiltonian. In another word, whether $\{\varphi_j, \phi_l\}$ span the valence band subspace of the new Hamiltonian without any conduction

band components. The question, however, is which Hamiltonian we shall use for this $N-s_l$ electron system. In the current paper, we are not deriving our orbital equation from an orbital dependent variational functional for both the Wannier function and the canonical orbital; as a result, the issue of which single particle Hamiltonian is not clear. The fixed ϕ_l is certainly not contained in the valence band subspace of the $N-s_l$ electron Hamiltonian $H_{\rm LDA}[N-s_l]$. However, it is not difficult to construct a single particle Hamiltonian for which the $\{\varphi_j, \phi_l\}$ constitutes its valence band subspace. For example, starting from $H_{\rm LDA}[N-s_l]$, we can always write down:

$$H_{\rm LDA}[N-s_l]\phi_l = \sum_j \beta_j \varphi_j + \alpha_l \phi_l + w_l \tag{S6}$$

Where $< w_l|\phi_l >= 0$ and $< w_l|\varphi_j >= 0$ (i.e, w_l is the residual). Then we can construct:

$$H_w = H_{\rm LDA}[N-s_l] - |w_l >< \phi_l| - |\phi_l >< w_l| \tag{S7}$$

It follows

$$H_w \phi_l = \sum_j \beta_j \varphi_j + \alpha_l \phi_l \tag{S8}$$

$$H_w \varphi_j = \beta_j \phi_l + \epsilon_j \varphi_j \tag{S9}$$

One can then easily re-diagonalize the Hamiltonian H_w using $\{\varphi_j, \phi_l\}$, and the eigen states will be the valence orbitals of H_w. In other words, $\{\varphi_j, \phi_l\}$ will span the valence band subspace of H_w. For small s_l, it is easy to show the H_w should have a gap, since when $s_l = 0$, H_w equals to $H_{\rm LDA}[N]$ that has a gap. From this argument, we realize that when we discuss the valence band subspace (during the process of removing electrons from a Wannier function), we need to be clear what the single particle Hamiltonian is. We like to emphasize that, at the current stage, we are treating the Wannier function non-self-consistently during the removing of electrons. We feel that even if a self-consistent treatment is used, the change for the Wannier function could be small. For example, if we take δ electrons from the canonical orbital (the usual ΔSCF approach), during the self-consistent calculation for the $N-\delta$ electron system, the most relaxation effect comes from the $N-1$ valence state, instead of the valence band maximum (VBM) state. Thus, even if we fix the VBM state (like we fix the Wannier function here), the error in $E(N-\delta)$ is usually rather small. Nevertheless, it might be interesting in the future to test the effects of the self-consistency during the removing of electrons. But even if $\phi_l(sl)$ depends on s_l, in Eq.(2) the ϕ_l must be $\phi_l(s_l = 0)$ when calculating band gap correction. Thus, all these might only change the procedure to calculate λ_l. If the major effect of the screening comes from the other $N-1$ electrons (as we discussed above), the self-consistent effects for ϕ_l should be quite small.

Supplementary Table

Supplementary Table S1: The calculated LDA, Wannier-corrected, and experimental band gaps (eV) of the 27 compounds. The most stable structures are adopted. For TiO_2, we adopt the rutile structure, and for SiC we adopt the zincblende structure.

Compounds	LDA	Wannier-corrected	Experiment
C	4.24	5.82	5.5
Si	0.51	1.11	1.12
Ge	0	0.63	0.7
SiC	1.34	2.69	2.42
AlN	4.33	6.35	6.19
AlP	1.46	2.44	2.5
AlAs	1.32	2.24	2.22
AlSb	0.90	1.63	1.61
GaN	1.73	3.47	3.48
GaP	1.42	2.33	2.35
GaAs	0.5	1.33	1.43
GaSb	-0.2	0.46	0.8
InN[a]	-0.4	1.0	0.9
InP	0.53	1.44	1.42
InAs	-0.5	0.35	0.41
InSb	-0.53	0.14	0.23
ZnO	0.66	3.41	3.44
ZnS	2.14	3.73	3.72
ZnSe	1.13	2.50	2.8
ZnTe	1.0	2.04	2.3
CdO[b]	-0.54	1.24	0.84
CdS	0.92	2.40	2.48
CdSe	0.28	1.55	1.73
CdTe	0.34	1.35	1.47
MgO	5.19	8.0	7.9
TiO_2	1.68	3.61	3.1
PbS	-0.32	0.67	0.4

[a]The experimental band gap in Ref. [S2] is obsolete for InN. Please refer to Ref. [S3].
[b]The experimental indirect (L$\rightarrow \Gamma$) band gap varies from 0.8 to 1.2 eV.

[S1] Perdew, J. P., Parr, R. G., Levy, M. & Balduz Jr., J. L. Density-functional theory for fractional particle number: Derivative discontinuities of the energy. *Phys. Rev. Lett.* **49**, 1691–1694 (1982).

[S2] Madelung, O. *Semiconductors: Data Handbook* (Springer, Berlin, 2004).

[S3] Davydov, V. Y. *et al.* Absorption and emission of hexagonal InN. Evidence of narrow fundamental band gap. *Phys. Stat. Sol. (b)* **229**, R1–R3 (2002).
